# Unveiling the long non-coding RNA profile of porcine reproductive and respiratory syndrome virus-infected porcine alveolar macrophages

**DOI:** 10.1186/s12864-021-07482-9

**Published:** 2021-03-12

**Authors:** Junxin Gao, Yu Pan, Yunfei Xu, Wenli Zhang, Lin Zhang, Xi Li, Zhijun Tian, Hongyan Chen, Yue Wang

**Affiliations:** State Key Laboratory of Veterinary Biotechnology, Heilongjiang Provincial Key Laboratory of Laboratory Animal and Comparative Medicine, Harbin Veterinary Research Institute, Chinese Academy of Agricultural Sciences, Harbin, China

**Keywords:** Porcine reproductive and respiratory syndrome virus, Long non-coding RNA, Porcine alveolar macrophage, mRNA-lncRNA correlation network

## Abstract

**Background:**

Long noncoding RNA (lncRNA) is highly associated with inflammatory response and virus-induced interferon production. By far the majority of studies have focused on the immune-related lncRNAs of mice and humans, but the function of lncRNAs in porcine immune cells are poorly understood. Porcine reproductive and respiratory syndrome virus (PRRSV) impairs local immune responses in the lungs of nursery and growing pigs, whereas the virus triggers the inflammatory responses. Porcine alveolar macrophage (PAM) is the primary target cell of PRRSV, thus PRRSV is used as an in vitro model of inflammation. Here, we profiled lncRNA and mRNA repertories from PRRSV-infected PAMs to explore the underlying mechanism of porcine lncRNAs in regulating host immune responses.

**Results:**

In this study, a total of 350 annotated lncRNAs and 1792 novel lncRNAs in PAMs were identified through RNA-seq analysis. Among them 86 differentially expressed (DE) lncRNAs and 406 DE protein-coding mRNAs were identified upon PRRSV incubation. GO category and KEGG pathway enrichment analyses revealed that these DE lncRNAs and mRNAs were mainly involved in inflammation- and pathogen infection-induced pathways. The results of dynamic correlated expression networks between lncRNAs and their predicted target genes uncovered that numerous lncRNAs, such as XLOC-022175, XLOC-019295, and XLOC-017089, were correlated with innate immune genes. Further analysis validated that these three lncRNAs were positively correlated with their predicted target genes including CXCL2, IFI6, and CD163. This study suggests that porcine lncRNAs affect immune responses against PRRSV infection through regulating their target genes in PAMs.

**Conclusion:**

This study provides both transcriptomic and epigenetic status of porcine macrophages. In response to PRRSV infection, comprehensive DE lncRNAs and mRNAs were profiled from PAMs. Co-expression analysis demonstrated that lncRNAs are emerging as the important modulators of immune gene activities through their critical influence upon PRRSV infection in porcine macrophages.

**Supplementary Information:**

The online version contains supplementary material available at 10.1186/s12864-021-07482-9.

## Background

Next-generation sequencing of host genomes has revealed that non-coding RNAs, including micro RNAs, lncRNAs, and circular RNAs, are transcribed from most genomes [1]. LncRNAs, defined as non-coding RNAs of more than 200 nucleotides in length, are most often endowed with polyadenylation, positioned in the nucleus, cytoplasm or both [2, 3]. Emerging evidence has shown that lncRNAs are involved in a variety of biological processes including genomic imprinting, cell proliferation and differentiation, and cellular developmental processes [4]. Transcriptome-wide studies have demonstrated that many lncRNAs exhibit highly tissue and cell type specific expression [5, 6], indicating that lncRNAs may be the driver of cell-specific response [7, 8]. For example, a terminal differentiation-induced lncRNA TINCR is specifically expressed in the late stage of human somatic tissue, which regulates the epidermal differentiation through TINCR-mRNA base-pairing interactions [9]. Several lncRNAs are tumor-specific, such as PVT1 [10] and HAGLROS [11], regulating the expression of oncogenes and tumor suppressor genes. LncRNA LOC646329, a cell proliferation regulator, appears low in neocortical tissues but enriches in radial glia cells [12]. These data indicated that lncRNAs may play important roles in lineage-specific differentiation or specialized cellular function.

The innate immune system is the first line of host defense against invading organisms. Recently lncRNAs have been identified as the key regulators in the innate immune response. One well-characterized lncRNA lincRNA-Cox2 has been identified to be a regulator of the transcription of macrophages, which can either activate the expression of IL-6 and IL-23a via the TLR2 pathway or inhibit the expression of IFN-stimulated genes (*IRF7*, *ISG15*, *IFI204*, and *OAS2*) [13]. Another immune-related lncRNA THRIL is highly induced in monocyte cell line THP-1, serving as an enhancer of TNFα and IL-6 through interacting with hnRNPL [14]. However, little is known regarding the function of lncRNAs in porcine innate immune cells during virus infection.

Porcine reproductive and respiratory syndrome virus (PRRSV) is the etiologic agent of porcine reproductive and respiratory syndrome, which is characterized by respiratory problem in growing pigs and reproductive failure in sows [15, 16]. Porcine alveolar macrophages (PAMs) are the primary target cells of PRRSV. Macrophages, as a group of innate immune cells, play a central role in monitoring viral infections including PRRSV, influenza A virus, and HIV. To successfully fight against PRRSV infection, macrophages have evolved various strategies to regulate antiviral responses, such as regulating the production of IFN-α/β [17]. The previous evidence suggested that PRRSV was susceptible to IFNs [18], whereas PRRSV-mediated suppression of IFNs production helped the virus circumvent the host antiviral responses [19]. Besides, the pro-inflammatory cytokines including IL-1, IL-6, IL-8, and TNF-α mainly produced in PAMs after virus invasion, play critical roles in infection and pathogenesis of PRRSV [20–23]. However, the intracellular regulatory mechanisms of lncRNAs related to these innate immune properties remain to be addressed. Here, we used PRRSV as an inflammatory model to stimulate PAMs and explored the regulatory role of porcine lncRNAs in innate immune responses.

## Results

### Expression profile of lncRNA in PAMs

**Fig. 1:**
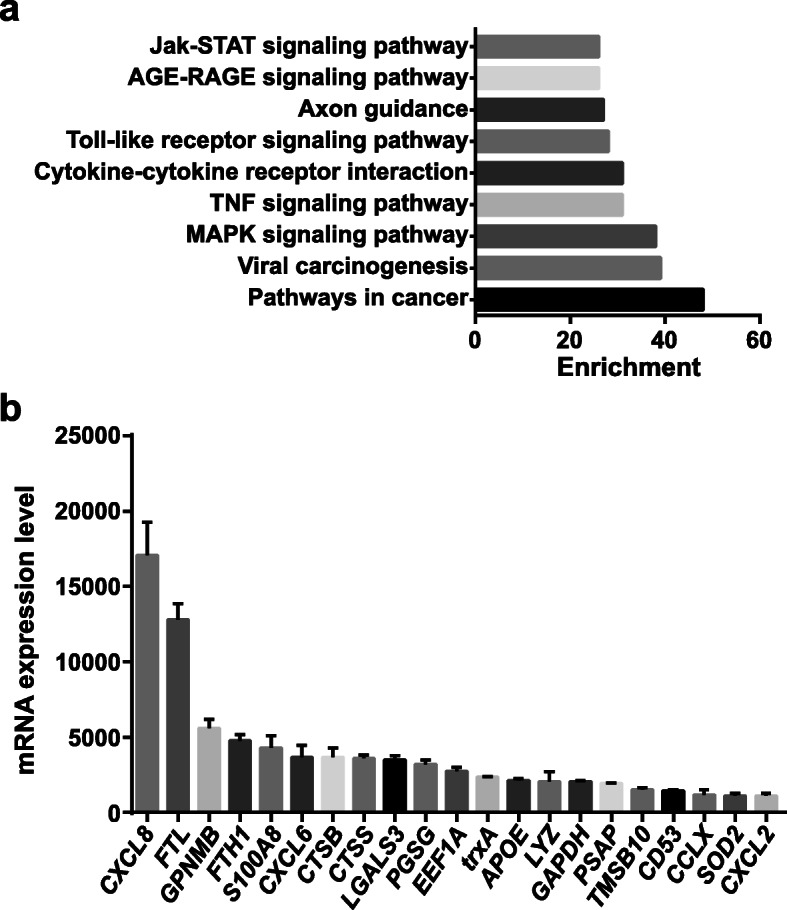
The expression profiles of endogenous lncRNAs and mRNAs in PAMs. **a** KEGG pathway analysis of lncRNAs in PAMs. **b** Top 20 abundant mRNAs in PAMs

To evaluate the performance of endogenous lncRNAs in PAMs, we designed a synthetic reference pool from three specific-pathogen-free (SPF) Landrace pigs by RNA-seq analysis. More specifically, our pool contained 2142 lncRNAs (350 known lncRNAs and 1792 novel lncRNAs) and 22,565 mRNAs. To predict the function of these lncRNAs, we performed KEGG pathway analysis of cis- and trans-regulated predicted mRNAs. The result showed that enriched GO and KEGG pathways were mainly related to the inflammatory response, such as “MAPK signaling pathway”, “cytokine-cytokine receptor interaction”, “TNF signaling pathway”, “toll-like receptor signaling pathway”, and “Jak-STAT signaling pathway” (Fig. 1a). To make a connection between the enriched lncRNAs and mRNAs, we screened out the top 20 abundant mRNAs. As shown in Fig. 1b, majority of the first 20 abundant mRNAs were inflammatory response-related genes, such as Chemokine (C-X-C motif) ligand 8 (*CXCL8*), Ferritin light chain (*FTL)*, *S100A8*, Chemokine (C-X-C motif) ligand 6 *(CXCL6)*, Cathepsin S *(CTSS)*, Galectin-3 *(LGALS3)*, Elongation factor 1-alpha 1 *(EEF1A)*, thioredoxin (*trxA*), Apolipoprotein E *(APOE)*, C-type lysozyme enzyme *(LYZ)*, Leukocyte surface antigen *(CD53)*, Chemokine ligand x *(CCLx)*, Superoxide dismutase 2 *(SOD2)*, and Chemokine (C-X-C motif) ligand 2 *(CXCL2)*. These data indicate that the PAMs are enriched in immune response-related lncRNAs and their predicted target genes, which allow them to respond quickly to invading organisms.

### Validation the susceptibility of PAMs to PRRSV infection

**Fig. 2:**
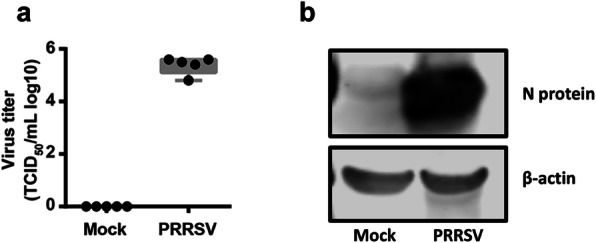
Validation of PAMs susceptibility to PRRSV infection. **a** The virus titer determined by TCID_50_. **b** The expression of viral N protein determined by Western-blotting

To perform the functional analysis of porcine lncRNAs, we established in vitro model of inflammation by incubating PAMs with a high-pathogenic PRRSV strain HuN4. Treated cells were then collected for RNA sequence analysis. To confirm whether the PAMs were successfully infected by PRRSV, virus replication was determined by TCID_50_ and Western blot assay. The results showed that the virus titer obtained from PAMs supernatant was 5.25 log10 TCID_50_/ml (Fig. 2a) and the expression of N protein was also confirmed in PRRSV-treated PAMs (Fig. 2b and Additional file 1).

### LncRNA expression profile in PRRSV-infected PAMs

**Fig. 3:**
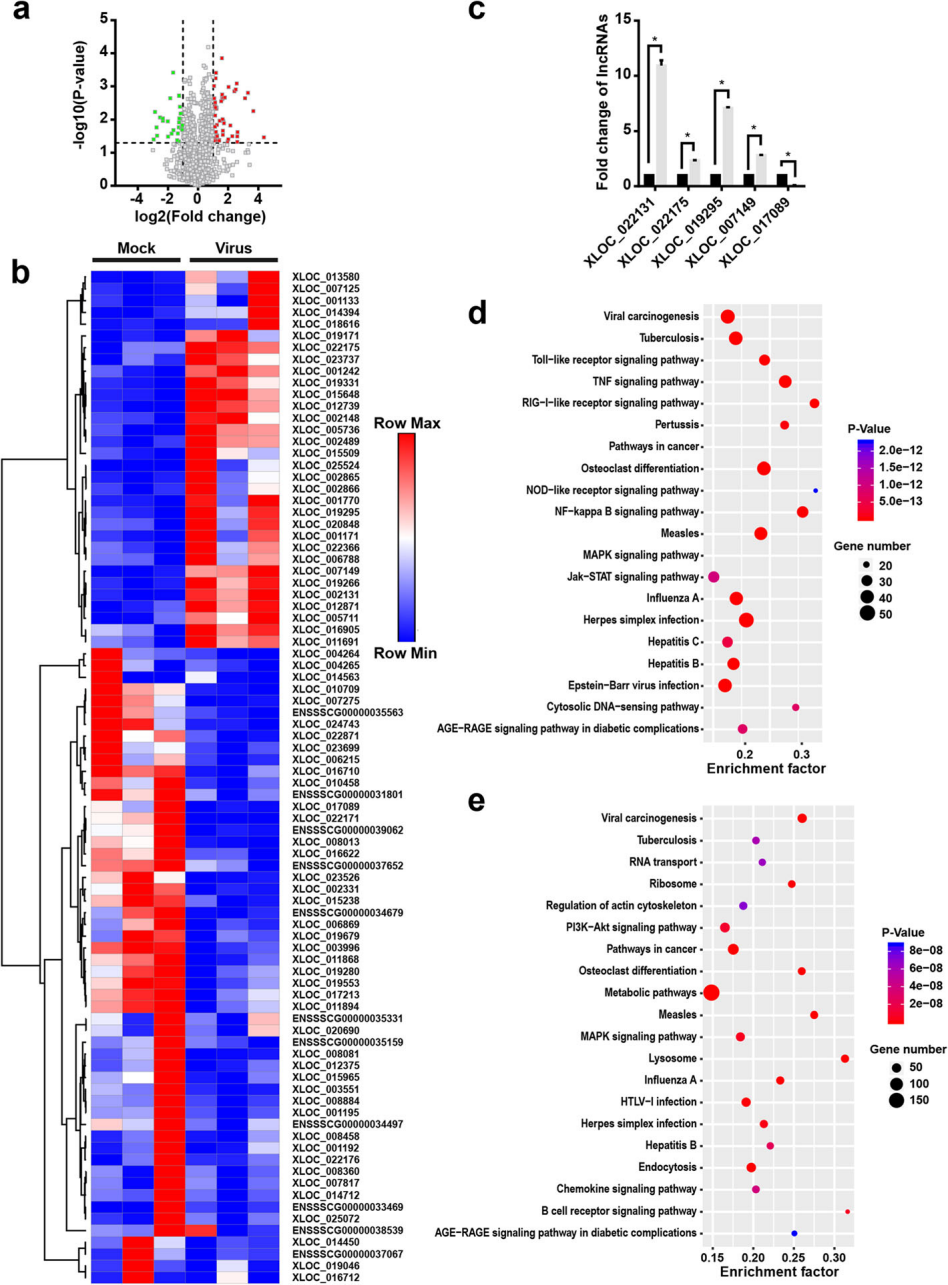
DE lncRNAs in PAMs upon PRRSV infection. **a** Volcano plots of DE lncRNAs between mock and PRRSV-infected PAMs. Red or green points represent the DE lncRNAs with statistical significance (*P* < 0.05). **b** Unsupervised hierarchical clustering and heat map of lncRNA expression between mock and PRRSV-infected PAMs. Each column represents a sample and each row represents a lncRNA. Red color indicates relatively high expression and blue color indicates relatively low expression. **c** Verification of lncRNAs expression in mock and PRRSV-infected PAMs. Expression levels of four upregulated and one downregulated lncRNAs were verified by qPCR and normalized to β-actin. The results were presented as the fold change to the corresponding uninfected control (*, *P* < 0.05). **d** and **e** Pathway analysis of DE lncRNAs after PRRSV infection. The dot plots represent the enrichment of the lncRNAs in each pathway. The color of each dot corresponds to the *P*-value. The size of each dot shows the number of lncRNAs. The horizontal axis represents the enrichment level of the pathways. A higher enrichment level means a larger change after PRRSV infection when the *P*-values are the same. Pathway enrichment of upregulated lncRNAs (**d**) and downregulated lncRNAs (**e**) was shown respectively. Results are representative of three independent experiments (means ± SD). *, *P* < 0.05. The *P* value was calculated using Student’s *t*-test

Freshly isolated PAMs were treated with PRRSV HuN4 or mock, and followed by RNA-seq methodology. The results showed that the expression levels of 86 relatively abundant lncRNAs (FPKM > 1) were significantly altered upon virus incubation (Fig. 3a and Additional file 2). Among them, 33 lncRNAs were upregulated and 53 were downregulated (fold change>2.0, *P*-value<0.05). To further employ the differentially expressed (DE) lncRNAs upon PRRSV infection, the unsupervised hierarchical clustering analysis was used. Heat maps showed overt self-segregated clusters in PAMs treated with PRRSV and mock (Fig. 3b). To further verify the accuracy of RNA-seq results, we tested four upregulated lncRNAs (XLOC-022131, XLOC-022175, XLOC-019295, and XLOC-007149) and one downregulated lncRNA (XLOC-017089) by quantitative PCR (qPCR). As shown in Fig. 3c and Table 1, XLOC-022131, XLOC-022175, XLOC-019295, and XLOC-007149 were significantly upregulated and XLOC-017089 was significantly downregulated, indicating the accuracy of RNA-seq data.

**Table 1:** Primers used for qPCR

Primer	Sequence (5′-3′)
Porcine -actin-F	CTTCCTGGGCATGGAGTCC
Porcine -actin-R	GGCGCGATGATCTTGATCTTC
Porcine-IFI6-F	GAAGACGCTCTGAGGACAAC
Porcine-IFI6-R	CGGTTGTGAAGCCCAGAG
Porcine-CXCL2-F	GGAAGTTTGTCTCAACCCCGC
Porcine-CXCL2-R	AGCCAGTAAGTTTCCTCCATCTC
Porcine-CD163-F	ATGGGCTAATTCCAGTGCAG
Porcine-CD163-R	GATCCATCTGAGCAAGTCACTCCA
XLOC-017089 -F	CTTAACCTACTGAGCCAAGCC
XLOC-017089-R	ATGTACTTTACCAGATTTGTCATGAAA
XLOC-022175-F	ACGAATAGTGAGTGTGAGGGC
XLOC-022175-R	GACAGAATGACTCTACTCACACG
XLOC-019295-F	GATCTCGTTGGGCTTCTCATAG
XLOC-019295-R	GAGCTTCCTCTGTCATACTTGG
XLOC-007149-F	CCTTGCTTCTGTTCTCCTGG
XLOC-007149-R	GTTCCTCATTCTCTTCCTCGG
XLOC-002131-F	GGTCTCCATGTCATTCCGATG
XLOC-002131-R	TTACTCACTTGCTCTGCCAC

We next performed GO and KEGG pathway analyses to evaluate the function of DE lncRNAs. As shown in Fig. 3d, the upregulated lncRNAs were primarily associated with “NF-κB signaling pathway”, “toll-like receptor signaling pathway”, “MAPK signaling pathway”, “RIG-I-like receptor signaling pathway”, “Jak-STAT signaling pathway”, and “TNF signaling pathway”. The downregulated lncRNAs were enriched in “PI3K-Akt signaling pathway”, “chemokine signaling pathway”, and “MAPK signaling pathway” (Fig. 3e). The GO and KEGG pathway patterns suggest that DE lncRNAs are largely associated with the inflammation- and pathogen infection-induced immune responses upon PRRSV infection, indicating that these DE lnRNAs may play critical roles in regulating the virus-induced inflammatory responses in PAMs.

### Gene expression signature in PRRSV-infected PAMs

**Fig. 4:**
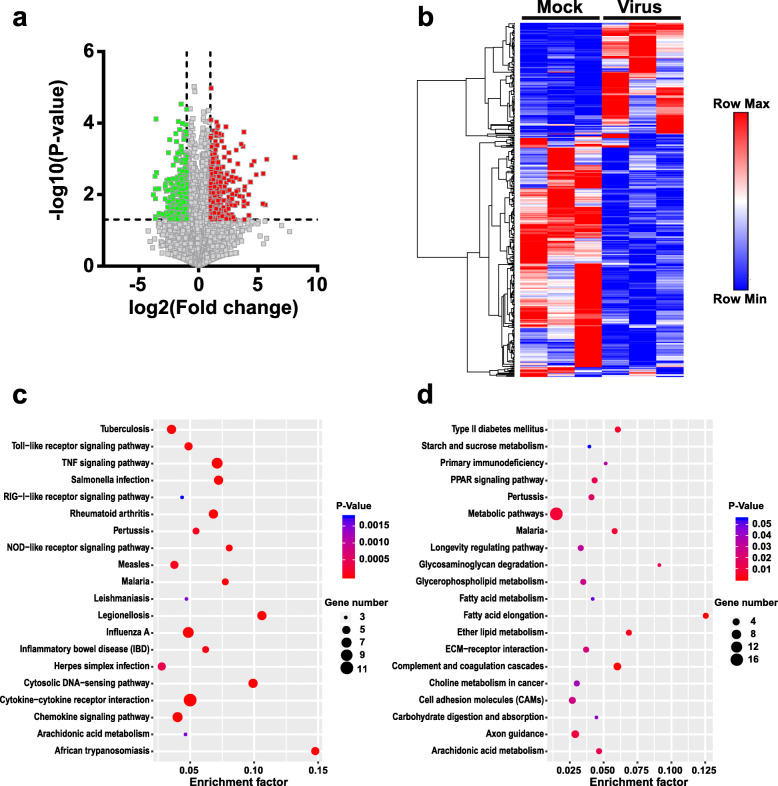
DE mRNAs in PAMs upon PRRSV infection. **a** Volcano plots of mRNAs between mock and PRRSV-infected PAMs. Red or green points represent the DE mRNAs with statistical significance (*P* < 0.05). **b** Unsupervised hierarchical clustering and heat map of mRNA expression in PAMs upon virus infection. Red color indicates upregulated expression and blue color indicates downregulated expression. Each column represents a PAM sample and each row represents an mRNA fragment. Mock represents mock-treated PAMs and virus represents PRRSV-infected cells. **c** and **d** Pathway analysis of DE mRNAs after PRRSV infection. The dot plots present the enrichment of the mRNAs in each pathway. Pathway enrichment of upregulated mRNAs (**c**) and downregulated mRNAs (**d**) was shown respectively

Based on the analysis of RNA-seq data, we also generated volcano plots to visualize the DE profile of protein-coding mRNAs. Totally 406 mRNAs were differentially expressed (126 upregulated and 280 downregulated mRNAs) in PAMs after PRRSV treatment (Fig. 4a and Additional file 3). The hierarchical clustering analysis revealed that DE mRNAs were precisely distinguished by PRRSV-infected and mock-treated PAMs (Fig. 4b). Furthermore, we performed the GO and KEGG pathway analyses for DE mRNAs. Figure 4c showed that the upregulated genes were enriched in “TNF signaling pathway”, “cytokine-cytokine receptor interaction”, “chemokine signaling pathway”, “rheumatoid arthritis”, “Toll-like receptor signaling pathway”, and “inflammatory bowel disease”. The most downregulated genes were enriched in pathways including “metabolic pathways” and “complement and coagulation cascades” (Fig. 4d). The pathway patterns determined by GO and KEGG analyses indicate that these DE genes are dominantly related to pathogen-induced inflammatory immune responses. Combined with lncRNA profiles, these results implicate that the DE lncRNAs may have indispensable regulatory functions in the DE genes in PAMs upon virus stimulation.

### Identification of the regulatory function of lncRNAs in immune responses

Correlation network is an innovative tool to better integrate the co-expressed genes with the regulatory functions of lncRNAs. Here the correlation analysis encompassed 86 lncRNA nodes and 406 mRNA nodes with significant changes in the virus-stimulated PAMs. By using a Pearson Correlation, we identified numerous sets of DE genes with temporal co-expression patterns (Additional file 4). To annotate the function of DE lncRNAs, we selected four immune-related genes including CXCL2, IFI6, IFITM1, and CD163 as modules to unearth the corresponding regulatory lncRNAs.

**Fig. 5:**
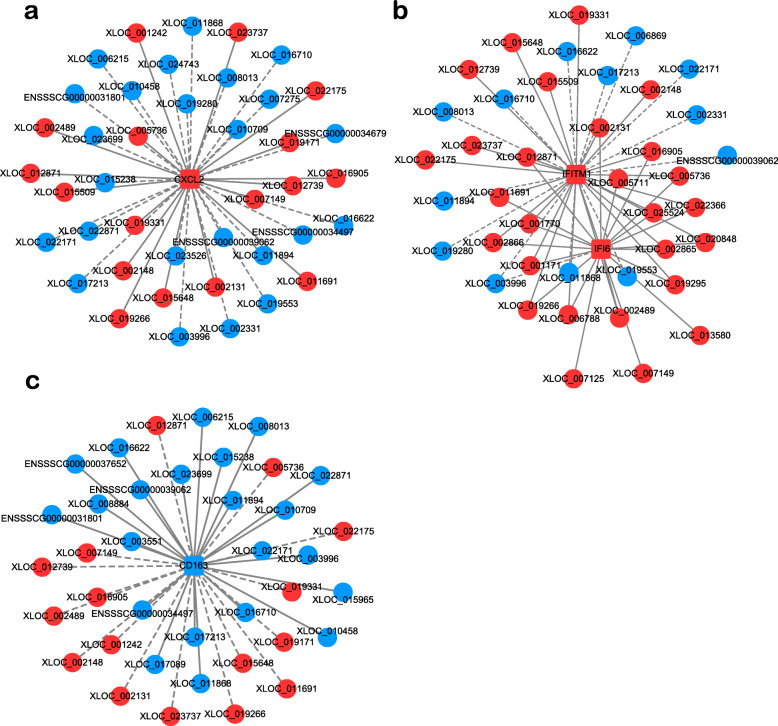
Correlation between lncRNAs and CXCL2, IFI6/IFITM1, and CD163 mRNA. The red color indicates the upregulated lncRNAs while the blue color indicates the downregulated lncRNAs. The solid lines represent positive correlation and the dash lines represent negative correlation. **a** CXCL2-lncRNAs network. **b** IFITM1/IFI6-lncRNAs network. **c** CD163-lncRNAs network

CXCL2, as a powerful chemoattractant, is exclusively secreted by monocytes and macrophages, involving in neutrophil recruitment and other immune responses during inflammation and wound healing [24]. For the CXCL2-lncRNAs network, 17 lncRNAs positively correlated with CXCL2 expression were upregulated, while 24 lncRNAs negatively correlated with CXCL2 expression were downregulated in PRRSV-infected cells (Fig. 5a). The CXCL2 module indicates that significantly enriched lncRNAs may play a dual role in the macrophages for microbial killing and initiating tissue repair [25].

Numerous IFNs stimulated genes, transcriptionally induced as the downstream signaling molecule of IFNs, can inhibit the infection of multiple viral families. Among these antiviral genes, the IFN-inducible protein (IFI) family and IFN-induced transmembrane protein (IFITM) family have broad-spectrum antiviral functions. The IFITM family consists of IFITM1, 2, 3, 5, and 6 [26], which can block the replication and infection of enveloped viruses [27]. IFI6 can target viral replication at the endoplasmic reticulum or distinct membranous organelles [28]. Here, the IFI6 and IFITM1 were selected to build the lncRNA/mRNA co-expression network. For the IFN-inducible genes network, 27 lncRNAs positively correlated with IFITM1/IFI6 expression were upregulated, while 13 lncRNAs negatively correlated with IFITM1/IFI6 expression were downregulated in PRRSV-infected cells (Fig. 5b). Our results indicate that the DE lncRNAs associated with IFN-inducible genes have the potential to regulate the antiviral functions in macrophages.

CD163 has been identified as the essential receptor that mediates PRRSV entry [29, 30]. Therefore, the CD163-lncRNAs correlation network was analyzed. As shown in Fig. 5c, 16 lncRNAs negatively correlated with CD163 expression were upregulated, while 22 lncRNAs positively correlated with CD163 expression were downregulated in PRRSV infected cells. The CD163 module suggests that DE lncRNAs may play important roles in PRRSV entry by regulating the expression level of CD163.

### Validation of differentially expressed lncRNAs by qPCR

**Fig. 6:**
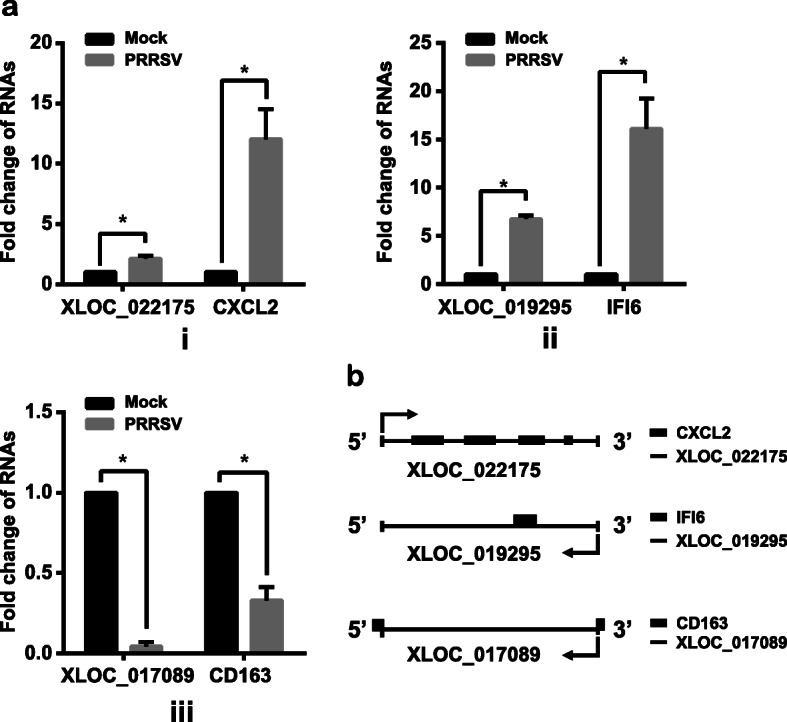
Verification of the correlation between lncRNAs and mRNAs. **a** PAMs were infected with PRRSV at a MOI of 5. At 24 hpi, total RNA was extracted and subjected to qPCR for detecting the expression levels of transcripts as indicated. (**i**) XLOC_022175 and CXCL2 mRNA. (**ii**) XLOC_019295 and IFI6 mRNA. (**iii**) XLOC_017089 and CD163 mRNA. **b** The location of lncRNAs and target mRNAs respectively. Results are representative of three independent experiments (means ± SD). *, *P* < 0.05. The *P* value was calculated using Student’s *t*-test

To verify our correlation networks, qPCR was used to examine three significantly differentially expressed lncRNA-mRNA pairs in PRRSV-infected PAMs. As shown in Fig. 6a and Table 1, all three lncRNA-mRNA pairs, including lncRNA XLOC-022175 vs CXCL2 (co-upregulated), XLOC-019295 vs IFI6 (co-upregulated), and XLOC-017089 vs CD163 (co-downregulated) exhibited significant changes after virus infection, which coincided with the predicted correlation networks. Additionally, the relationship between these three lncRNAs and their neighboring genes was analyzed based on the genomic location and transcriptome expression profile (Fig. 6b and Table 2). Sequence analysis revealed that XLOC-22175 as a sense lncRNA located at the CXCL2 genomic locus, suggesting that the upregulated XLOC-022175 may promote the expression of CXCL2 (Fig. 6a i). Sequence databases also showed that both XLOC-019295 and XLOC-017089 were antisense lncRNAs, and located near the genomic region of either IFI6 or CD163, indicating that both lncRNAs may facilitate the expression of IFI6 and CD163, respectively (Fig. 6a ii and iii). Interestingly, XLOC-019295 was predicted to be a trans-regulatory element for the expression of distant gene IFITM1 (Table 2). Although the correlations of these three lncRNA-mRNA pairs were confirmed by qPCR, their potential immune regulatory function needs further investigation.

**Table 2:** LncRNA-mRNA pairs with Pearson Correlation coefficients > 0.9

LncRNA	Correlation coefficients.	Target gene	KEGG name	Function
XLOC_022175	0.949422683	ENSSSCG00000008959	CXCL2	cis
XLOC_019295	0.997191011	ENSSSCG00000034570	IFI6	cis
	0.941706488	ENSSSCG00000014565	IFITM1	trans
XLOC_017089	0.952479684	ENSSSCG00000033146	CD163	cis

## Discussion

There are numerous lncRNAs available in public databases concerning their sequence, expression, and function [31–33]. The recent growth of next-generation sequencing technology has accelerated the research progress of lncRNAs. Although tens of thousands of lncRNAs have been discovered, only a small fraction of lncRNAs have been found to regulate gene expression in mouse and human. Even more noteworthy is the fact that only 15% of mouse lncRNAs are expressed in human and vice versa, suggesting dramatic species-specificity [34]. However, there are few databases about porcine lncRNAs and their potential functions [35–38], resulting in majority of porcine lncRNAs remains to be unidentified. Macrophages, as one of the critical regulators of both normal homeostasis and pathology, have attracted much attention from researchers to investigate the molecular mechanisms responsible for their function. In this study, the high-throughput RNA-seq method was utilized to investigate the detailed information about lncRNAs in porcine macrophages. We found 2142 lncRNAs abundantly expressed in PAMs. To describe the biological functions of these lncRNAs, GO and KEGG pathways are commonly introduced [39, 40]. GO and KEGG pathway analyses revealed that the regulatory signaling pathways of these lncRNAs mainly enriched in IFNs and cytokines production, indicating that lncRNAs may have a tight connection with immune response in porcine macrophages.

To further determine the regulatory function of these lncRNAs, we incubated PAMs with PRRSV to induce the inflammation model [41]. According to RNA-seq data, we defined a set of 86 DE lncRNAs in PAMs after PRRSV infection. GO and KEGG pathway enrichment analyses revealed that most DE lncRNAs and protein-coding genes were associated with inflammatory pathways in PAMs. Since the network analysis can provide a global view of all possible lncRNAs-mRNA expression associations based on the different immune background, here, the network analysis was used to predict the functional annotations of DE lncRNAs. We observed the cooperative expression relationships between four protein-coding genes (CXCL2, IFI6, IFITM1, and CD163) and their predicted regulator lncRNAs. Likewise, other studies have also employed a similar strategy to identify functional lncRNA signatures in different types of cancer [42, 43].

LncRNAs are recognized as transcriptional regulators that may be involved in cis or trans regulation of genes located in their vicinity or at distant loci [44]. In cis, the sense and antisense lncRNAs interact with genes transcribed from the same DNA region; whereas in trans, the interaction is with genes located at distant loci or even at other chromosomes [45, 46]. Here, after validation by qPCR, three selected lncRNAs were showed to have a positively correlated expression with their predicted target genes in PAMs. By using sequence alignment between full-length lncRNAs and nearby mRNAs, we especially considered protein coding gene loci which act as hosts for lncRNA transcripts. In other published literature about lncRNA profiles upon PRRSV infection, there is no such detailed information provided [37, 38, 47, 48]. Only Zeng et al. revealed that DE lncRNA XR_297549.1 induced by PRRSV infection was predicted to both cis-regulate and trans-regulate its neighboring gene, prostaglandin-endoperoxide synthase 2 [49]. In this study, XLOC-022175, a sense lncRNA in cis-regulatory fashion, was predicted to be positively correlated with CXCL2, which is mainly released from activated macrophages and capable of attracting CXCR2+ cells, such as neutrophils to sites of inflammation [50]. Our data also showed that IFI6 and IFITM1 were predicted to be the cis- and trans-regulated target genes of XLOC-019295, respectively. Both IFI6 and IFITM1 belong to IFN-stimulated genes with antiviral activity [26]. IFITM1, a virus-restriction factor, have been proved to restrict the replication of several viruses, including PRRSV, PRV, PCV2, and SIV [51–53]. XLOC-017089 was identified as an antisense lncRNA and predicted to act in cis to regulate CD163 expression, which has been determined to be the essential receptor for PRRSV [16, 54]. These presented data suggest that our proposed method can better reveal the function of lncRNAs not only through the correlation network of different expression but also through cis or trans regulatory mode of lncRNAs located. By using the similar methodology, other cutting-edge studies have indicated that lncRNAs regulate the function of their target genes through cis- and trans-acting, such as Xist, PTEN, and HOTAIR [43, 55, 56].

Taken together, our findings provide novel insights on functional characterization of lncRNAs in innate immune responses of porcine macrophages. However, the detail mechanisms of these core lncRNAs need to be further studied and established. We need precisely characterize these lncRNAs phenotypes and identify its functions during in vitro and in vivo infection. Technically we need conduct genetic modification systems to answer whether lncRNAs XLOC-022175, XLOC-019295, and XLOC-017089 play critical roles in inflammation, antiviral response, and PRRSV infection. More specifically, we will investigate whether lncRNA XLOC-022175 can regulate CXCL2 secretion to recruit neutrophils to sites of PRRSV infection, whether XLOC-019295 plays a duel role in host defense against infectious diseases, and whether XLOC-017089 can modify PRRSV infection through regulating the expression of the scavenger receptor CD163.

## Conclusion

In summary, RNA-seq can be a useful tool to analyze the expression profiles of lncRNAs and their regulatory function. Here, the transcriptome analysis revealed that a total of 86 DE lncRNAs and 406 DE protein-coding mRNAs were mainly involved in PRRSV-induced inflammation and immune responses in PAMs. Correlated expression network analysis identified that numerous lncRNAs participated in the innate immune responses. Correlation analysis of DE lncRNAs revealed that the predicted target genes of XLOC-022175, XLOC-019295, and XLOC-017089 were related to the inflammation and antiviral signaling pathways. These DE lncRNAs comprised a valuable atlas that could be used to connect lncRNAs expression with viral pathogenesis. Further studies are required to investigate the regulatory roles of these lncRNAs during virus infection.

## Methods

A full step-by-step process can be found in Additional file 5.

### Animals, cells and viruses

Three five-week-old SPF Landrace pigs were from Harbin Veterinary Research Institute, Chinese Academy of Agricultural Sciences. Pigs were euthanized by Pentobarbital sodium intravenously (100 mg/kg) and sacrificed. Immediately, lungs were collected sterilely and primary PAMs were freshly isolated. Animal experiments were approved by the Animal Care and Use Committee of Harbin Veterinary Research Institute of Chinese Academy of Agricultural Sciences (Approval ID: 200720–01) and experiments were performed according to the regulations and guidelines established by this committee. PAMs were cultured in DMEM (Life Technologies, USA) supplemented with 10% heat-inactivated FBS (Hyclone), 100 U/ml penicillin, 100 μg/mL streptomycin at 37 °C in a 5% CO_2_ incubator (Thermo Scientific, USA). Marc-145 cells, a monkey kidney cell line, were maintained in DMEM as well. The PRRSV North American-like strain HuN4 (GenBank accession number EF635006), a high pathogenic PRRSV strain [57] was cultured in Marc-145 cells and PAMs as indicated.

### TCID_50_ assay

Median tissue culture infectious does (TCID50) assay was performed on Marc-145 cells as previously described [57]. Virus samples were collected by centrifugation for 10 min at 8000×g and diluted by 10-fold serial dilution. Cell monolayers were inoculated with diluted viruses and cultured for 5–6 days until the appearance of cytopathic effect. The virus titers were calculated by Reed & Muench method.

### Western blotting

Western blotting analysis was performed as described previously with a slight modification [58]. PAMs were inoculated with PRRSV or mock at a MOI of 5. At 24 h postinfection, cells were lysed with Pierce IP lysis buffer (Thermo Scientific, Rockford, IL). The cell lysates were separated by SDS-PAGE under reducing condition and transferred onto PVDF membrane. After blocking, the membrane was incubated with the appropriate primary and secondary antibodies. The membranes were scanned and analyzed using an Odyssey instrument (Li-Cor Biosciences). The blotting mAb against PRRSV nucleocapsid (N) protein was prepared in our laboratory. Anti-actin mAb (C4) was purchased from Santa Cruz Biotechnology (Santa Cruz, CA). The IRDye-conjugate secondary antibody was from Li-Cor Biosciences (Lincoln, NE).

### Total RNA extraction and quantitative PCR

Total RNA was extracted from cells with TRIzol (Invitrogen, SIGMA), and was then reverse transcribed into cDNA using PrimeScript RT reagent Kit with gDNA Eraser by random primers (TaKaRa, Japan) according to the manufacturer’s instructions. Quantitative PCR (qPCR) was carried out in a QuantStudio 5 system (Applied Biosystems) using SYBR premix Ex Taq (TaKaRa, Japan). Fold changes were determined using the cycle threshold (ΔΔCT) method [59].

### Whole transcriptome library preparation and sequencing

RNA transcripts were purified from total RNA with oligo (dT)-attached magnetic beads and fragmented into short fragments. First-strand cDNA and second-strand cDNA were subsequently synthesized using random hexamer primer. The cDNA fragments were purified and resolved with EB buffer for terminal repair, the addition of single nucleotide A and adapters. After size selecting and retrieving by AMPure XP beads, the products were used for PCR amplification to obtain the library. Agilent 2100 Bioanalyzer and ABI StepOnePlus Real-Time PCR System were used to assess the qualification and quantification of those libraries. The eligible libraries were sequenced using Illumina HiSeqTM X TEN.

### RNA-seq data analysis

Clean reads were obtained by removing reads with adaptors, reads with unknown bases (N bases more than 5%), and low quality reads (bases qualities lower than 10). Next, HISAT [60] tools were used to map clean reads to the indexed reference transcriptome. Gene expression levels were normalized using FPKM method by RSEM [61, 62]. Differentially expressed genes were screened by Edge R [63] with the criteria of fold change ≥2 and FDR ≤ 0.01.

### Function prediction of lncRNAs

To determine the expression profile of lncRNAs, RNA transcripts were reconstructed and identified by mapped reads using StringTie [64] and cuffcompare [65]. The known lncRNAs were acquired by comparing the assembled transcripts with annotated lncRNAs from NCBI, Ensembl, and UCSC. To predict new lncRNAs, we only retained transcripts longer than 200 nucleotides, more than one exon, and optimum expression threshold of FPKM > 0.5 in at least one sample. The software CNCI [66], CPC [67], and txCdsPredict were used to predict the protein-coding potential of new transcripts with default parameters. Only transcripts that did not pass the protein-coding score test (CPC score < 0, CNCI score < 0, txCdsPredict score < 500) were predicted as new lncRNAs. The expression level of lncRNA was calculated using FPKM within the software RSEM. Transcripts with an FDR < 0.01 and fold change ≥2 were identified as significantly DE lncRNAs using EdgeR. The potential target genes of DE lncRNAs in cis- and trans-regulatory effects were predicted. The neighboring protein-coding genes were predicted as the cis target genes. To predict trans target genes, according to the FPKM values of the different expression of lncRNAs and mRNAs in all the samples, the Pearson Correlation coefficient between the lncRNAs and the mRNAs were calculated, and the threshold for positive correlation was set to Pearson Correlation > 0.8.

### Differential expression analysis of lncRNAs and mRNAs

For each sample, FPKM was a normalized estimation of both mRNA and lncRNA based on RNA-seq data. FPKM was calculated from the number of reads that mapped to each particular gene sequence taking into account the gene length and the sequencing depth. By using DESeq [68] package in R language and *t*-test, mRNA and lncRNA differential expression analyses were performed for all pairwise comparisons between PRRSV-infected groups and mock-treated groups. A corrected *P*-value < 0.05 by Student’s *t*-test with Benjamini-Hochberg FDR adjustment was used as the cut-off for significantly DE genes.

### Heatmap and functional annotation of differentially expressed transcripts

To show fold changes of the DE transcripts between groups, hierarchical heatmaps were clustered by the one minus Pearson Correlation method and generated by MORPHEUS (https://software.broadinstitute.org/morpheus). To gain insight into the functions of DE transcripts, the associated GO terms were identified using DAVID Bioinformatics Resources (version 6.7, http://david.abcc.ncifcrf.gov/). Specifically, GeneRatio and BgRation were used to classify the GO category. DAVID software was used to analyze the signaling pathways of DE genes according to KEGG. Two-sided Fisher’s exact test and x^2^ test were used to select significant pathways, and the FDR was calculated to correct the *P*-value.

### LncRNA-mRNA correlation network

The lncRNA-mRNA correlation network was constructed by correlation calculation based on the values of the normalized signal intensity of specific expression in the DE genes. For each pair of lncRNA-mRNA, the Pearson Correlation was calculated to determine the significance of correlation and the correlation value cut-off was 0.8. Correlation degrees of lncRNAs and mRNAs were calculated by counting their correlated counterparts. The *P*-value denoted the significant level of gene co-expression and the threshold of significance was *P*-value < 0.05.

### Statistical analysis

For next-generation sequencing data, all the experimental condition was independently repeated three times and in each of three biological repetitions, three technical replicas were made. Clean reads were analyzed by Edge R and the criteria of fold change ≥2 and FDR ≤ 0.01. Correlation network data were calculated by Pearson Correlation coefficient and the correlation value cut-off was 0.8 and the threshold of significance was *P*-value < 0.05. All statistical data were expressed as mean ± standard deviation (SD) of three independent experiments and analyzed using Student’s *t*-test. A *P*-value of < 0.05 was considered statistically significant.

## Supplementary Information

**Additional file 1.**

**Additional file 2.**

**Additional file 3.**

**Additional file 4.**

**Additional file 5.**

## Data Availability

The RNA-seq data supporting the conclusions has been deposited into the NCBI Sequence Read Archive (SRA), under the accession number SRP278343 (https://www.ncbi.nlm.nih.gov/sra/?term=SRP278343).

## Abbreviations

APOE: Apolipoprotein E
CCL: Chemokine ligand
CD53: Leukocyte surface antigen
CD163: Scavenger receptor
circRNAs: Circular RNAs
CTSS: Cathepsin S
CXCL: Chemokine (C-X-C motif) ligand
CXCR: Chemokine (C-X-C motif) receptor
DE: Differently expressed
EEF1A: Elongation factor 1-alpha 1
FDR: False discovery rate
FPKM: Fragments Per Kiolbase MillionTPM
FTL: Ferritin light chain
GO: Gene ontology
hnRNP: Heterogeneous nuclear ribonucleoprotein
hpi: Hours post-incubation
IFI: IFN-inducible protein
IFITM: IFN-induced transmembrane protein
IFN: Interferon
IL: Interleukin
IRF: Interferon regulatory factor
ISG: Interferon-stimulated gene
KEGG: Kyoto Encyclopedia of Genes and Genomes
LGALS3: Galectin-3
LncRNA: Long noncoding RNA
LYZ: C-type lysozyme enzyme
miRNA: Micro RNA
MOI: Multiplicity of infection
OAS2: 2′-5′ oligoadenylate synthase
PAM: Porcine alveolar macrophage
PRRSV: Porcine reproductive and respiratory syndrome virus
SD: Standard deviation
SOD2: Superoxide dismutase 2
SPF: Specific Pathogen Free
TCID_50_: 50% tissue culture infective dose
TNFα: Tumor necrosis factor
trxA: Thioredoxin
