# Supplementary material for: Unveiling the long non-coding RNA profile of porcine reproductive and respiratory syndrome virus-infected porcine alveolar macrophages
Source: BMC Genomics. 2021 Mar 12;22:177. doi: 10.1186/s12864-021-07482-9 (PMC7953715; doi:10.1186/s12864-021-07482-9)
Supplement: Supplementary file 1 — Additional file 1. [file 12864_2021_7482_MOESM1_ESM.pptx]

## Slide 1
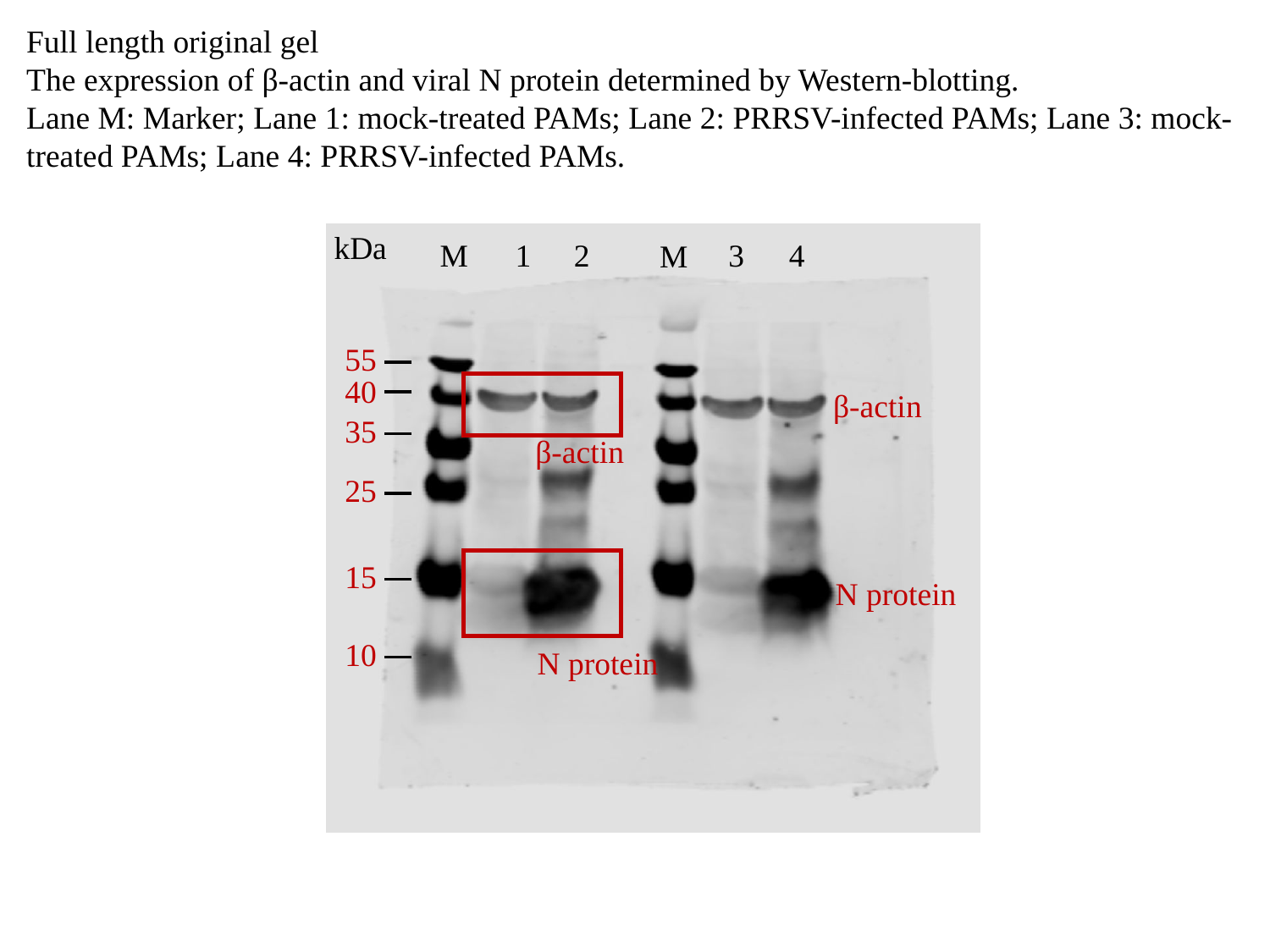

Full length original gel
The expression of β-actin and viral N protein determined by Western-blotting.
Lane M: Marker; Lane 1: mock-treated PAMs; Lane 2: PRRSV-infected PAMs; Lane 3: mock-treated PAMs; Lane 4: PRRSV-infected PAMs.
kDa
M
1
2
3
4
M
55
40
35
25
15
10
β-actin
β-actin
N protein
N protein
