# Supplementary material for: Unveiling the long non-coding RNA profile of porcine reproductive and respiratory syndrome virus-infected porcine alveolar macrophages
Source: BMC Genomics. 2021 Mar 12;22:177. doi: 10.1186/s12864-021-07482-9 (PMC7953715; doi:10.1186/s12864-021-07482-9)
Supplement: Supplementary file 5 — Additional file 5. [file 12864_2021_7482_MOESM5_ESM.docx]

**Protocol for porcine long non-coding RNA profile**

**Planning**

**Pigs**

Three five-week-old SPF Landrace pigs were from Harbin Veterinary Research Institute, Chinese Academy of Agricultural Sciences. Animal experiments were approved by the Animal Care and Use Committee of Harbin Veterinary Research Institute of Chinese Academy of Agricultural Sciences (Approval ID: 200720-01) and experiments were performed according to the regulations and guidelines established by this committee.

**Experiments**

Experimental planning can ensure that the minimum number of animals is used in experiments, but also do not waste time and reagents. The term power is commonly used with reference to all sample size estimations in research. There is no data for power calculations, thus pilot studies are carried out. The study plan outlining the lncRNAs profile experiment can be seen in **Figure A**.

**Experimental procedure**

**Collection of porcine lungs**

TIMING: Day 0, ~4.0 hour.

1. Three five-week-old SPF Landrace pigs were from Specific Pathogen Free (SPF) facility of Harbin Veterinary Research Institute, Chinese Academy of Agricultural Sciences. The facility uses an environment that is free of certain (not all) infectious organisms that are pathogenic and/or capable of interfering with research objectives to breed pigs. Before selecting the experimental pigs, appropriate samples were collected for diagnostic test to avoid the presence of zoonotic disease or other diseases of concern to population health.
2. Collection time was between 8:30 am to 12:30 pm. The collection order was randomized, with each animal at a different time on one day.
3. Personal protective equipment and precautions were used during handling pigs. There were two professional assistants for intravenous injection and binding the pigs to ensure a smooth euthanization procedure. Pigs were euthanized by Pentobarbital sodium intravenously (100 mg/kg) and sacrificed. Before the lung was collected, pigs should be confirmed painless death without vital signs (unconscious and brain-dead: absence of corneal reflex, absence of eyelash reflex, and absence of rhythmic breathing).
4. Each lung was collected sterilely and left on ice.
5. Once the lung was collected, the animal carcass disposal was handled properly to protect public health in accordance with laboratory instructions and the local law.

**Collection of porcine alveolar macrophages**

TIMING: Day 0, ~3.0 hours.

1. The lung was gently washed with ~500 mL DMEM three times in clean bench, and porcine alveolar macrophages (PAMs) were then collected by centrifugation at 1,000×g for 10 min. Cells were then counted and suspended in DMEM at the concentration of 1×10^^7^ cells per mL.
2. Partial cells from each pig were randomly divided into three groups (0.35 mL cell suspension per group). Total RNA was immediately extracted from cells with TRIzol and subjected to next generation sequencing.
3. Partial PAMs were cultured in 6-well-plate at a density of 3.5×10^^6^ cells per well in 2 mL of DMEM supplemented with 10% heat-inactivated FBS, 100 U/ml penicillin, and 100 μg/mL streptomycin at 37°C in a 5% CO_2_ incubator. Cells from each pig were plated in 6 wells and cultured for overnight.

**PAMs incubation with PRRSV**

TIMING: Day 1, ~1.5 hours.

1. After washing, PAM monolayers from each pig were randomly divided into three groups. Cells were then incubated with either mock control or PRRSV at a MOI of 5.
2. After 60 min of incubation, cell monolayers were washed and then further cultured in DMEM supplemented with 2% FBS and antibiotics.

**Collection of treated PAMs**

TIMING: Day 2, ~2.0 hours.

1. At 24 hours after virus inoculation, treated cells were collected by centrifugation.
2. Total RNA was extracted from cells with TRIzol.

**Next generation sequencing and data analysis**

TIMING: Day 3-.

A process about analyses of next generation sequencing can be seen in **Figure B**.

**Figure A:**


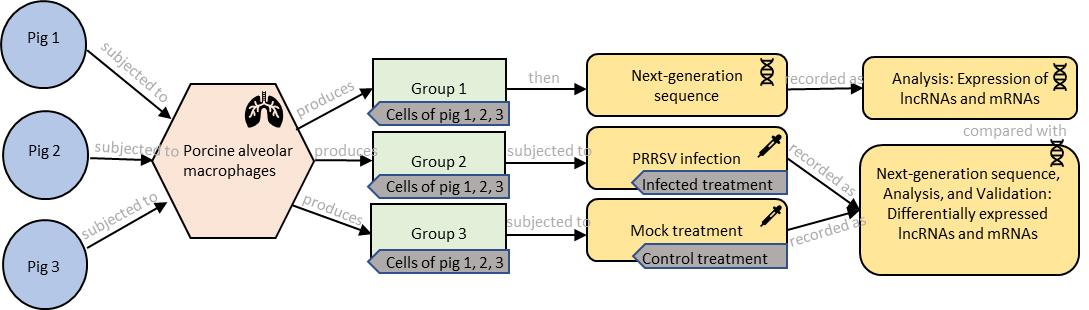


This flow chart shows the outlining the lncRNAs profile experiment based on the Next-generation sequence. Each experimental condition was independently repeated three times and in each of these three biological repetitions, three technical replicas were made. Clean reads were obtained by removing reads with adaptors, reads with unknown bases, and low quality reads. Clean reads were analysed by Edge R to explore the effect of porcine differentially expressed lncRNAs and mRNAs in regulating host immune responses (Edge R with the criteria of fold change ≥ 2 and FDR ≤ 0.01).

**Timing**


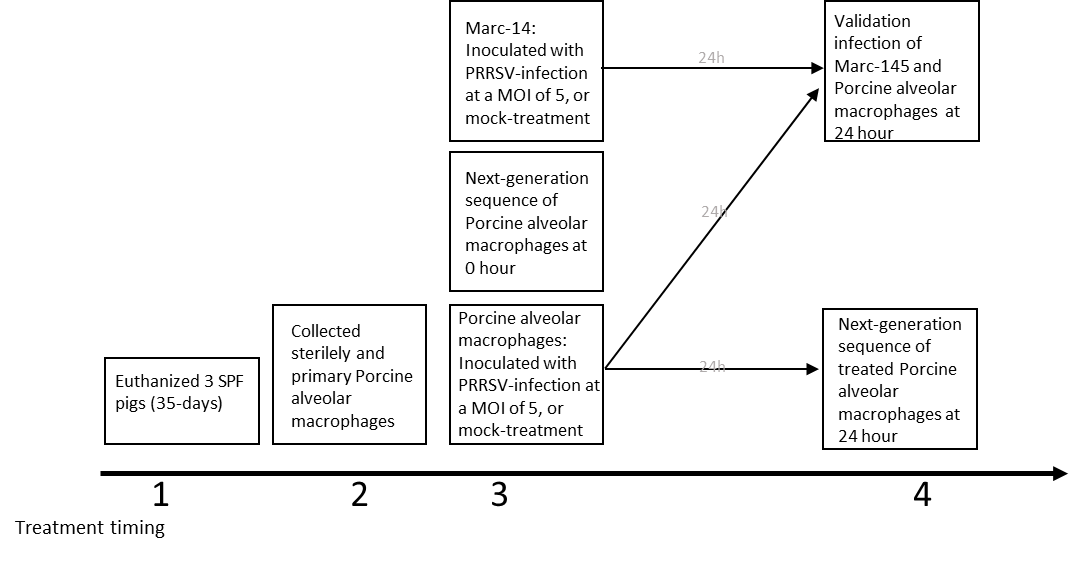


**Figure B:**


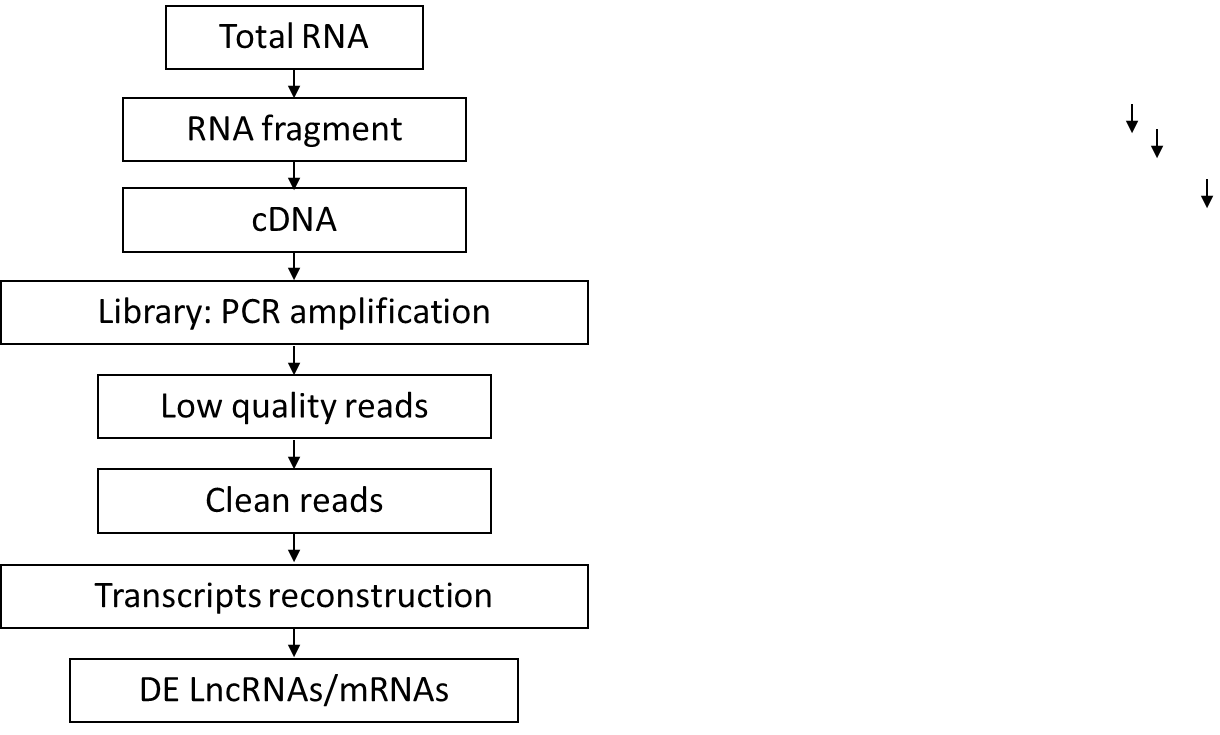


Detailed workflow of lncRNAs/mRNAs sequencing
